# The Polyphenol Fisetin Protects Bone by Repressing NF-κB and MKP-1-Dependent Signaling Pathways in Osteoclasts

**DOI:** 10.1371/journal.pone.0068388

**Published:** 2013-07-04

**Authors:** Laurent Léotoing, Fabien Wauquier, Jérôme Guicheux, Elisabeth Miot-Noirault, Yohann Wittrant, Véronique Coxam

**Affiliations:** 1 Clermont Université, Université d'Auvergne, Clermont-Ferrand, France; 2 INRA, UMR 1019, Unité de Nutrition Humaine, CRNH Auvergne, Clermont-Ferrand, France; 3 Université de Nantes, UFR Odontologie, Nantes, France; 4 NSERM, UMRS 791, LIOAD, Nantes, France; 5 INSERM, UMR 990, Clermont-Ferrand, France; Universidade Federal do Rio de Janeiro, Brazil

## Abstract

Osteoporosis is a bone pathology leading to increase fractures risk and challenging quality of life. Since current treatments could exhibit deleterious side effects, the use of food compounds derived from plants represents a promising innovative alternative due to their potential therapeutic and preventive activities against human diseases. In this study, we investigated the ability of the polyphenol fisetin to counter osteoporosis and analyzed the cellular and molecular mechanisms involved. *In vivo*, fisetin consumption significantly prevented bone loss in estrogen deficiency and inflammation mice osteoporosis models. Indeed, bone mineral density, micro-architecture parameters and bone markers were positively modulated by fisetin. Consistent with *in vivo* results, we showed that fisetin represses RANKL-induced osteoclast differentiation and activity as demonstrated by an inhibition of multinucleated cells formation, TRAP activity and differentiation genes expression. The signaling pathways NF-κB, p38 MAPK, JNK and the key transcription factors c-Fos and NFATc1 expressions induced by RANKL, were negatively regulated by fisetin. We further showed that fisetin inhibits the constitutive proteasomal degradation of MKP-1, the phosphatase that deactivates p38 and JNK. Consistently, using shRNA stable cell lines, we demonstrated that impairment of MKP-1 decreases fisetin potency. Taken together, these results strongly support that fisetin should be further considered as a bone protective agent.

## Introduction

Bone homeostasis results from highly regulated activities of bone-forming osteoblasts and bone-resorbing osteoclasts. Imbalance between these two cell types leads to osteoporosis, a disease characterized by a low bone mineral density and an impaired bone microarchitecture leading to subsequent increased risk of fractures. Osteoblasts and osteoclasts differentiation and activity are tightly dependent upon cytokines, growth factors and hormones which activate complex signaling networks, specific transcription factors and the induction of their target genes involved in the differentiation process.

Osteoclasts are multinucleated giant cells derived from hematopoietic progenitors of the monocyte/macrophage lineage through a differentiation process mainly governed by two key cytokines: macrophage colony-stimulating factor (M-CSF) and receptor activator of nuclear factor-κB ligand (RANKL) [1], [2]. M-CSF induces the proliferation of osteoclasts precursors cells, sustains their survival and stimulates the expression of RANK, the receptor of RANKL [3], [4]. The interaction between RANK and RANKL leads to the recruitment of TNF receptor-associated factor 6 (TRAF6) and the subsequent activation of several downstream signaling pathways including the nuclear factor κB (NF-κB) as well as the p38 mitogen-activated protein kinase (MAPK), the c-jun-N-terminal kinase (JNK) and extracellular signal-regulated kinase (ERK) [5], [6], [7], [8]. NF-κB induction is a critical event as the genetic disruption of nfκb1 and nfκb2 genes coding for p50 and p52 transcription factors in mice leads to osteopetrosis due to impaired osteoclast differentiation [9], [10], [11]. It participates in the early induction of the transcription factor nuclear factor of activated T cells 1 (NFATc1), a master gene of osteoclast differentiation [12], [13]. The activation of MAPKs results in the phosphorylation of c-jun and its association with c-Fos to form the essential AP-1 transcription factor also involved in NFATc1 induction [14], [15]. NFATc1 then regulates the transcription of several target genes such as calcitonin receptor (CTR), tartrate resistante acid phosphatase (TRAP), matrix metalloproteinase 9 (MMP9) or cathepsin K that participate in osteoclast phenotype and bone matrix degradation [2], [16].

Fisetin, a flavonoid polyphenol present in plants, fruits and vegetables [17] has been described as a potent natural molecule with multiple beneficial biological activities including inhibition of prostate cancer growth [18], neuroprotection [19], [20] or prevention of rheumatoid arthritis [21]. Moreover, *in vitro* experiments reveal that fisetin exhibits anti-inflammatory activities by counteracting the NF-κB signaling pathway in lipopolysaccharide (LPS) treated macrophages [22], which share a common origin with osteoclasts. As inflammation exerts a critical role in post-menopausal and pathological osteoporosis [23], [24], we hypothesized that fisetin may counter bone loss in estrogen deficiency and inflammation-induced osteoporosis mice models. *In vivo*, we report that fisetin significantly prevents both ovariectomy and LPS-induced bone loss. *In vitro*, we further analyzed the cellular and molecular associated mechanisms and demonstrated that fisetin decreases RANKL-induced osteoclast differentiation by inhibiting NF-κB, p38 and JNK signaling.

## Materials and Methods

### Ethics Statement

All animal procedures were approved by the institution’s animal welfare committee (Comité d’Ethique en Matière d’Expérimentation Animale Auvergne: CEMEAA) and were conducted in accordance with the European’s guidelines for the care and use of laboratory animals (2010-63UE). Animals were housed in the animal facility of the INRA Research for Human Nutrition (Agreement N°: C6334514) (http://www1.clermont.inra.fr/unh/telechargementinternet/ienplaquette.pdf). All surgeries were performed under anesthesia, and all efforts were made to minimize suffering.

### Antibodies and Reagents

The antibodies were purchased from Cell Signaling (Danvers, MA, USA: IKKα, IKKβ, phospho IKKα/β Ser176/180, IκBα, phospho-IκBα Ser32-36, p65, phospho-p65 Ser536, p38, phospho-p38 Thr180/Tyr182, JNK, phospho-JNK Thr183/Tyr185, c-jun, phospho-c-jun Ser63, p42/p44, phospho-p42/p44 Thr202/Tyr204, c-Fos and HA), Santa Cruz Biotechnology (Santa Cruz, CA, USA: MKP-1, β-actin) and Roche Diagnostics (Meylan, France: c-Myc). Reagents were purchased from Sigma-Aldrich (St Louis, MO, USA : fisetin - ref. F4043, LPS serotype 026:B6, hydroxypropyl cellulose, N-Ethylmaleimide), Calbiochem (Darmstadt, Germany: MG132), R&D Systems Europe Ltd (Abingdon, UK: RANKL) and Extrasynthese (Genay, France: fisetin - ref. 1167 S).

### Plasmid Constructs

Expression vector for myc-tagged MKP-1 was generated by subcloning the murine full lengh MKP-1 cDNA into the pCMV-Myc vector (Zhenlin Li, Université Pierre et Marie Curie, Paris, France). The 3xκB-Luc-SV40 reporter was obtained from Jiake Xu (University of Western Australia, Nedlands, Australia) and the HA-Ub wt from Claudine Pique (Institut Cochin, Paris, France).

### Cell Culture and Transfections

Primary bone marrow cultures cells (BMC) for osteoclast formation were isolated from the femurs and tibiae of 3 to 5 week-old C57/BL6 female mice. Raw264.7 osteoclast precursors [25] were obtained from ATCC (Manassas, VA, USA). BMC cells and Raw264.7 were cultured in α-MEM (Invitrogen, Carlsbad, CA, USA) supplemented with 10% heat-inactivated FBS and 100 units/ml penicillin-streptomycin. For transfections, 10^6^ Raw264.7 in 6 wells plate were transfected with 0.5–1 µg of DNA using jetPrime (Polyplus Transfection, Illkirch Germany) in OPTI-MEM (Invitrogen).

### Establishment of shRNA-Raw264.7

Control and MKP-1 shRNA vectors were stably introduced in Raw264.7 using lentiviral particules (Sigma-Aldrich) followed by puromycin selection (4 µg/ml).

### Luciferase Reporter Assays

Raw264.7 were transfected with pCMV-β-Gal and NF-κB-luc reporter for 6 hours, pretreated with DMSO as control or fisetin for 3 hours then with RANKL and DMSO or fisetin for 48 additional hours. Firefly luciferase activity was determined using the Luciferase Assay System (Promega, Fitchburg, WI, USA) according to the manufacturer’s instructions and the RLU were related to the total protein concentration for each point. Transfection efficiency was monitored using β-Gal measurement. Experiments were conducted three times in triplicate.

### Cell Viability

Raw264.7 cells were seeded at a density of 3500 cells/well in 96 wells plate for 12 hours then treated with DMSO as control or fisetin (1 to 10 µM) for 48 hours in α-MEM supplemented with 2% FBS and antibiotics. The cell proliferation/viability was determined using an XTT based method (Cell Proliferation Kit II, Sigma-Aldrich) according to the supplier's recommendations. The coloration was measured using a microplate reader at 450 nm with a 650 nm reference wavelength.

### TRAP Staining and Activity

For TRAP staining and activity, 2*10^5^ BMC cells or Raw264.7 cells were seeded in 35 mm dishes for 12 hours, then treated with DMSO as control or fisetin (1 to 10 µM) for 3 hours then with DMSO or fisetin (1 to 10 µM) and RANKL (50 ng/ml) for 7 (BMC) or 4 (Raw264.7) days. Cell culture medium with RANKL and fisetin was changed every two days. TRAP staining was performed using the leukocyte acid phosphatase kit (Sigma-Aldrich) according to the supplier's recommendations following cell fixation. The multinucleated cells (more than 3 nuclei) were counted. For TRAP activity, cells were lysed in high salt buffer (0.4 M NaCl, 25 mM Hepes pH7.7, 1.5 mM MgCl2, 0.2 mM EDTA, 1%NP40 supplemented with proteases inhibitor cocktail). Lysates were incubated at 37°C in p-nitrophenyl phosphate buffer (125 mM sodium acetate buffer pH 5.2, 100 mM p-nitrophenyl phosphate, 1 mM L(+) sodium tartrate) and the absorbance was assessed in kinetic using a microplate reader at 405 nm. For each sample, protein concentration was analyzed to express the mean OD/minute/milligram of protein.

### RT-qPCR

Cell and bone total RNA extractions were performed using TRIzol reagent (Invitrogen), RT were performed using cDNA DyNamo Synthesis kit (Thermo Scientific, Waltham, MA, USA) and Real-time PCR analysis were carried out with Mastercycler ep Realplex2 (Eppendorf, Hamburg, Germany). All values were normalized to the level of GAPDH mRNA. Primers sequences are:

Cathepsin K: 5′-CGAAAAGAGCCTAGCGAACA-3′, 5′-TGGGTAGCAGCAGAAACTTG-3′

c-Fos: 5′-GGGACAGCCTTTCCTACTACC-3′, 5′-GATCTGCGCAAAAGTCCTGT-3′

CTR: 5′-TGCGAGGGGATCTATCTTCA-3′, 5′-GTTGGCACTATCGGGAACC-3′

GAPDH: 5′-AACTTTGGCATTGTGGAAGG-3′, 5′-GGATGCAGGGATGATGTTCT-3′

IκBα: 5′-ACGAGCAAATGGTGAAGGAG-3′, 5′-ATGATTGCCAAGTGCAGGA-3′

MCP-1∶5′-CCAGCACCAGCACCAGCCAA-3′, 5′-TGGGGCGTTAACTGCATCTGGC-3′

MIP-1α: 5′-GCTCCCAGCCAGGTGTCATTTTCC-3′, 5′-GGGGTTCCTCGCTGCCTCCA-3′

MKP-1∶5′-TGGTTCAACGAGGCTATTGAC-3′, 5′-GGCAATGAACAAACACTCTCC-3′

MMP9∶5′-CGACATAGACGGCATCCAG-3′, 5′-CTGTCGGCTGTGGTTCAGT-3′

NFATc1∶5′-CACATCCCACAGCCCAAT-3′, 5′-TCTGTGCTCTGCTTCTCCAC-3′

RANTES: 5′-TGCAGAGGACTCTGAGACAGC-3′, 5′-GAGTGGTGTCCGAGCCATA-3′

TRAP: 5′-CCAGCGACAAGAGGTTCC-3′, 5′-AGAGACGTTGCCAAGGTGAT-3′

### Ubiquitination Assay

Myc-MKP-1 and Ub-HA plasmids were transfected in Raw264.7 cells. The following day, cells were treated with DMSO as control or 5 µM fisetin for 4 hours and the assays were conducted as described previously [26]. Briefly, cells were lysed for 20 min on ice in ubiquitination assay buffer (150 mM NaCl, 50 mM Tris-HCl pH 7.5, 1% deoxycholic acid, 1% NP-40, 50 µM MgCl2, 0.1% SDS, supplemented with 10 mM N-Ethylmaleimide, 50 µM MG132, 10 mM p-nitrophenyl phosphate disodium salt, 20 mM β-glycerol phosphate, 100 µM Na3VO4, 1 mM PMSF, and 1X complete protease inhibitor cocktail), after which SDS concentration was adjusted to 1%, and whole cell extracts boiled for 10 minutes before being diluted 10-fold with ubiquitination assay buffer without SDS. These extracts (500 µg) were subjected to overnight immunoprecipitation with c-Myc antibody (2.5 µg) and immunoblotting.

### Animal Studies

Throughout the study, animals were housed in a controlled environment (12∶12 h light-dark cycle, 20–22°C, 50–60% relative humidity/1 mouse per plastic cage with free access to water. C57BL/6J mice were delivered to our facility 2 weeks before the study for acclimatization to our animal environment and semi-purified standard diet. For all the experiments, the 8 weeks old mice (n = 12/group) were orally given by gavage each day vehicle (1% ethanol, 0.2% hydroxypropyl cellulose in distilled water) as control or with fisetin (5 to 50 mg/kg body weight in vehicle) for one week. Then, for the OVX experiment, the mice were either sham operated or bilaterally ovariectomized and received either vehicle or fisetin (5 to 25 mg/kg body weight in vehicle) by gavage each day for 4 weeks. At the end of the experiment, uterine horns, tibias, femurs and blood samples were collected for analysis. For the LPS induced bone loss experiment, vehicle (PBS) or LPS (Serotype 026:B6, 25 mg/kg body weight) were injected subcutaneously once a week for 3 weeks on mice calvariae under anesthesia and received either vehicle or fisetin (5 to 50 mg/kg body weight in vehicle) by gavage each day during the 3 weeks. At the end of the experiment, tibias, femurs, liver, spleen and blood samples were collected for analysis. Same experiments were conducted and stopped 24 hours after the first LPS injection and the femurs were collected for RNA extraction and qRT-PCR analysis.

### BMD Analysis

Bone morphological analysis was performed using an eXplore CT 120 scanner (GE Healthcare, Little Chalfont, United Kingdom). After removing soft tissues, left femurs were placed in PBS buffer with 10% formaldehyde at 4°C for one week and scanned. Acquisition consisted of 360 views acquired in 1° increments collected in one full gantry rotation, with a 20 ms exposure/view and X-ray tube settings being 100 kV and 50 mA. CT images were reconstructed using a modified conebeam algorithm with an isotropic voxel of 0.045 × 0.045 × 0.045 mm3. CT scans were analyzed using MicroView® version 2.3 software (GE Healthcare). A hydroxyapatite calibration phantom (SB3, Gamex RMI, WI) was used to convert gray-scale levels to HA density values. Trabecular bone of the distal femur was selected for bone mineral density and bone volume fraction (BVF = Bone Volume/Total volume) analyses by fitting a cylindrical region (r = 0.7 mm) of interest in the center of the femur, starting 0.1 mm proximally from the growth plate and extending a further 0.32 mm in the proximal direction. Bone mineral density was estimated as the mean converted gray-scale level within the region of interest.

### Bone Micro-architecture Analysis

To perform a measurement, the specimen was mounted on a turntable that could be shifted automatically in the axial direction (angular step: 0.675°/reconstruction angular range: 186.30°). Aluminum filter (0.5 mm thick) was placed between X-rays source and sample. Micro-architecture (secondary spongiosa) was analyzed using X-ray radiation micro-CT SkyScan 1072 (BRUKER-MICROCT, Kontich, Belgium). Pictures of 1024*1024 pixels were obtained using 37 kV and 215 µA. According to camera settings final pixels measured 5.664 µm leading to a voxel of 1.817*10^−7^ mm^3^. Calculation of histomorphometric parameters was performed using CTAn ® and Nrecon® softwares (version 1.11. and 1.6.1.7 respectively).

### Serum CTX-1, Osteocalcin and sTNFRI Measurements

Serum CTX-1 levels, a specific marker of bone resorption, were determined using a mouse-specific ELISA assay according to the manufacturer’s protocol (Immunodiagnostic Systems EURL, Paris, France). Serum osteocalcin levels, a specific marker of bone formation, were measured using a mouse osteocalcin ELISA assay according to the manufacturer’s protocol (Biomedical Technologies Inc, Stoughton, MA, USA). Serum sTNFRI levels, a marker of inflammation, were assessed using a mouse-specific ELISA assay according to the manufacturer’s protocols (R&D).

### Statistical Analysis

Results are expressed as mean ± SD. Data were analyzed either by ANOVA Fisher’s (2 groups) or Newman’s Kell (more than 2 groups) test using XLSTAT (Addinsoft, Paris, France). Results were considered significant for p<0.05.

## Results

### Fisetin Significantly Prevents Ovariectomy-induced Bone Loss

**Figure 1:**
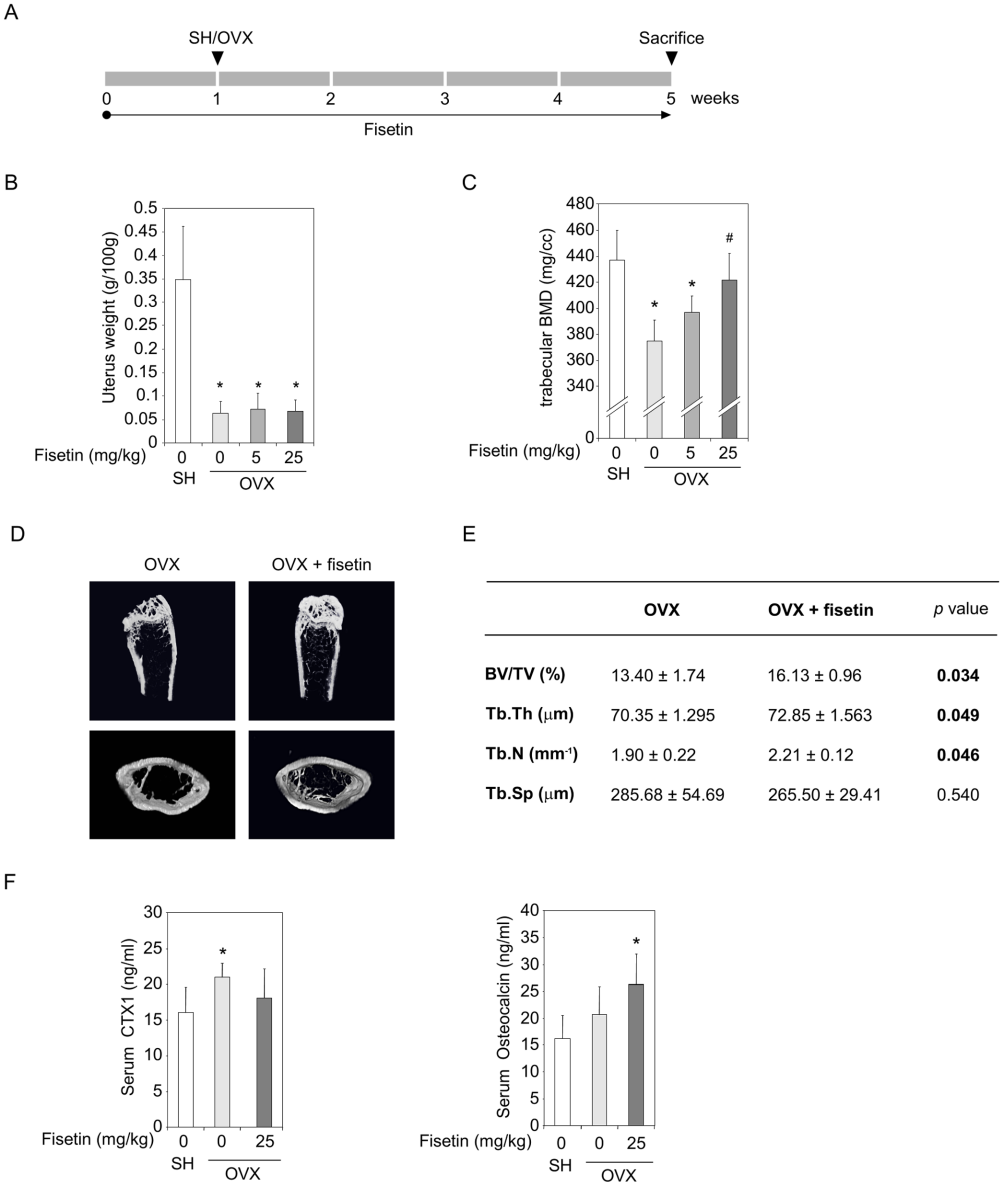
Fisetin significantly prevents ovariectomy-induced bone loss. (A). Study design. One week prior ovariectomy, mice (n = 12/group) received by gavage vehicle or fisetin at 5 and 25 mg/kg. The animals were subjected to sham operation (SH) or ovariectomy (OVX), then vehicle or fisetin was administrated by gavage for 4 weeks. At the end of the experiment, the uterus were weighed (B), the femurs were analyzed for trabecular bone mineral density (BMD) (C) and micro-architecture (D and E: OVX and OVX+fisetin 25 mg/kg). BV/TV: bone volume/total volume, Tb.Th: trabecular thickness, Tb.N: trabecular number, Tb.Sp: trabecular spaces. (F). Serum CTX1 and osteocalcin were analyzed by ELISA. For all data, (*) significantly different from SH, p<0.05, (#) significantly different from OVX-fisetin 0 mg/kg, p<0.05.

In order to investigate the impact of fisetin on bone *in vivo*, we used a well described mice model of bone loss induced by estrogen deficiency to mimic postmenopausal osteoporosis. Mice were administrated orally by gavage different doses of fisetin for one week, then were either sham operated (SH) or subjected to ovariectomy (OVX) and administrated orally by gavage different doses of fisetin for 4 weeks (Fig. 1A). The ovariectomy was validated by the atrophy of the uterine hornes at the end of the experiment (Fig. 1B). As expected, osteopenia was demonstrated in OVX mice as shown by a significant lower trabecular bone mineral density (BMD) than in the SH mice (Fig. 1C). Interestingly, the trabecular BMD of the OVX mice that received 25 mg/kg of fisetin was significantly higher than the one of the control OVX mice. Using µCT analysis, we confirmed this bone sparing effect of fisetin as the OVX mice fed with the polyphenol (25 mg/kg) had increased bone volume/total volume (BV/TV) ratio, trabecular thickness (Tb.Th) and trabecular number (Tb.N) (Fig. 1D and 1E). Finally, the serum CTX1 concentration, a bone resorption marker, tended to be lower in the fisetin fed OVX group compared to the control OVX group (Fig. 1F). Inversely, in the OVX-fisetin group, osteocalcin, reflecting osteoblast activity, was even higher than in the SH group. These data demonstrate that fisetin significantly prevents ovariectomy-induced bone loss.

### Fisetin Significantly Counters Inflammation-induced Bone Loss

**Figure 2:**
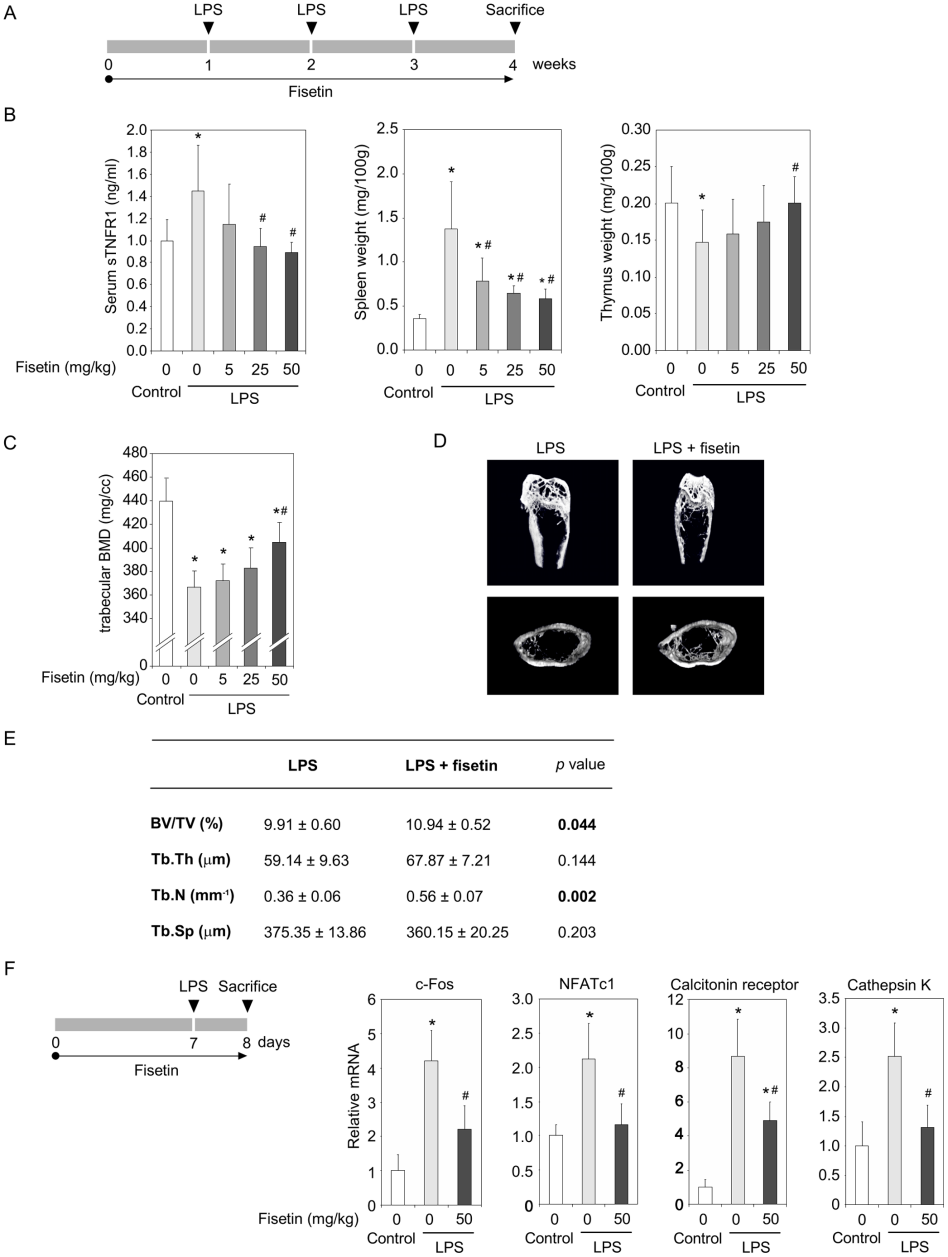
Fisetin significantly counters inflammation-induced bone loss. (A). Study design. One week before LPS injection, mice (n = 12/group) received by gavage vehicle or fisetin at 5, 25 and 50 mg/kg. Vehicle (PBS) or lipopolysaccharide (LPS –25 mg/kg) was injected subcutaneously once a week for 3 weeks on the calvariae of mice receiving by gavage vehicle or fisetin at 5, 25 and 50 mg/kg. (B). At the end of the experiment, serum sTNFR1 was measured by ELISA and the spleen and thymus were weighed. The femurs were analyzed for trabecular BMD (C) and micro-architecture (D and E: LPS and LPS+fisetin 50 mg/kg). (F). Similar experiments were performed and stopped 24 hours after the first LPS injection. The femurs were collected for transcriptomic analysis. For all data, (*) significantly different from control, p<0.05, (#) significantly different from LPS-fisetin 0 mg/kg, p<0.05.

Fisetin has been shown to exert anti-inflammatory actions *in vivo* [21]. Thus, we studied the potential of fisetin in a mice inflammation-induced bone loss model (Fig. 2A) [27]. As expected, lipopolysaccharide (LPS) injection induced an increase of the serum soluble TNF receptor 1 (sTNFR1) level and the spleen weight, and an atrophy of the thymus as already described [28] (Fig. 2B). All these parameters were significantly and dose-dependently reversed by the administration of fisetin by gavage. Concerning bone health, the 50 mg/kg dose of fisetin prevented bone loss induced by inflammation. Indeed, trabecular BMD (Fig. 2C), BV/TV and trabecular number were significantly higher in mice fed with the 50 mg/kg dose of fisetin than in LPS mice (Fig. 2D and 2E). Interestingly, fisetin tended to correct the early disruption of gene expression profile in bones after 24 hours following LPS injection (Fig. 2F). Indeed, early osteoclastic markers such as c-Fos, NFATc1, calcitonin receptor and cathepsin K were significantly reduced in the LPS-fisetin group as compared to the LPS control group. These results confirm the bone protective effect of fisetin *in vivo* and suggest that it could control osteoclast physiology.

### Fisetin Represses RANKL-induced Osteoclast Differentiation

**Figure 3:**
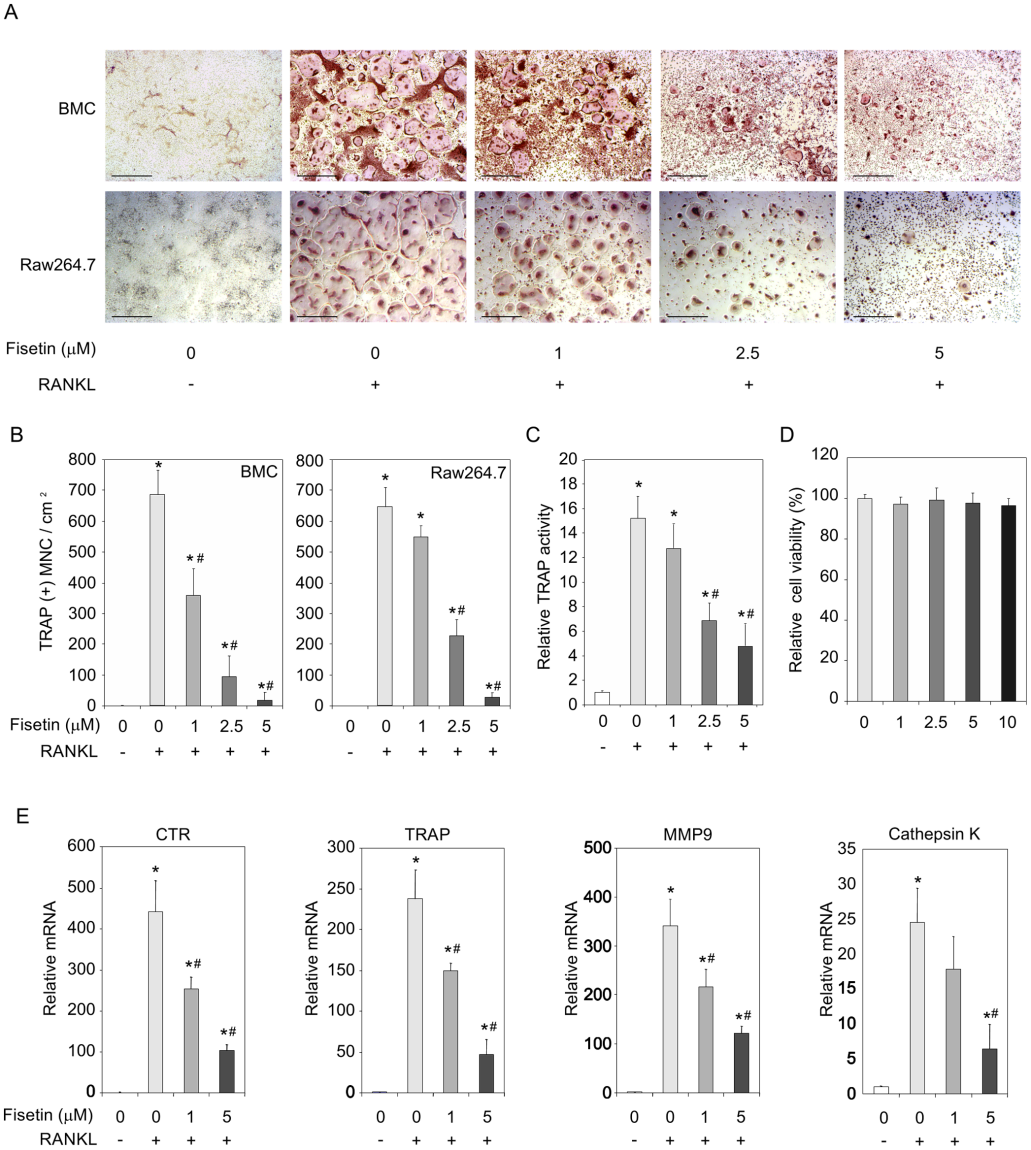
Fisetin represses RANKL-induced osteoclast differentiation. (A). Primary bone marrow cultures cells (BMC) and osteoclasts precursors Raw264.7 were pre-incubated with DMSO as control (fisetin 0 µM) or different doses of fisetin (1 to 5 µM) for 3 hours, then induced to differentiate in the presence of RANKL and DMSO as control (fisetin 0 µM) or fisetin (1 to 5 µM). After, 7 days (BMC) or 4 days (Raw264.7), TRAP staining was performed. Scale bars correspond to 500 µm. (n = 3 wells, representative of 3 independent experiments). (B). Giant TRAP (+) multinucleated cells (MNC: more than 3 nuclei) were counted at the end of the differentiation process. (C). Raw264.7 TRAP activity was measured. (n = 3 wells, representative of 3 independent experiments). (D). Osteoclast precursors Raw264.7 were cultured for 48 hours in the presence of DMSO as control (fisetin 0 µM) or different doses of fisetin (1 to 5 µM) and the relative viability was measured by an XTT assay. (n = 8 wells, representative of 3 independent experiments). (E). Indicated mRNAs of Raw264.7 were analyzed by RT qPCR after a terminal differentiation process performed as in A. (n = 3 wells, representative of 3 independent experiments). For all data, (*) significantly different from control, p<0.05, (#) significantly different from RANKL-fisetin 0 µM, p<0.05.

To evaluate how fisetin may control osteoclast physiology, we investigated its action *in vitro* on primary bone marrow cultures cells (BMC) and osteoclast precursors Raw264.7 differentiation and activity. After 7 days of culture in the presence of RANKL, the BMC differentiated in TRAP (+) multinucleated cells (MNC) as revealed by a TRAP staining (Fig. 3A, upper images and 3B, left panel). Interestingly, the presence of fisetin resulted in a dose dependent inhibition of this process. A similar result was observed in Raw264.7 cultures after 4 days of differentiation with RANKL (Fig. 3A, lower images and 3B, right panel). The enzymatic TRAP activity measured at the end of the differentiation process was also repressed by fisetin in Raw264.7 (Fig. 3C). These effects could not be attributed to a decrease of cell viability by fisetin (Fig. 3D). The expression of the osteoclastic differentiation mRNAs CTR, TRAP, MMP9 and cathepsin K induced by RANKL, were significantly lowered by fisetin (Fig. 3E), confirming the repressive potential of fisetin on osteoclast differentiation.

### Fisetin Inhibits RANKL-induced NF-κB Activity

**Figure 4:**
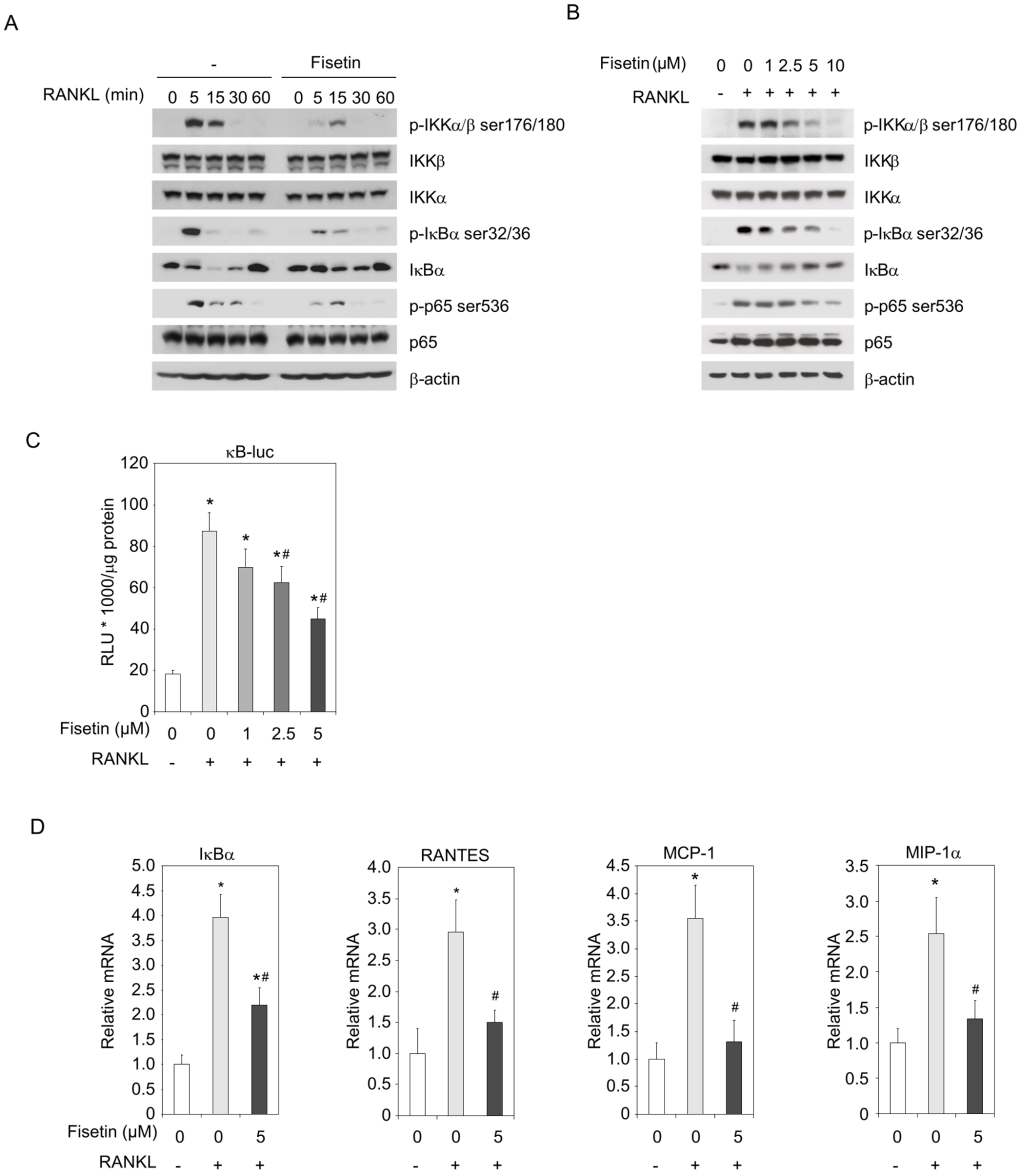
Fisetin inhibits RANKL-induced NF-κB activity. (A). Raw264.7 osteoclast precursors were pre-incubated with DMSO as control (-) or fisetin (5 µM) for 3 hours, then induced for 5 to 60 minutes with RANKL in the presence of DMSO as control (-) or fisetin (5 µM). Total protein extracts were analyzed by western-blotting for the indicated proteins. Blots are representative of 3 independent experiments. (B). Similar experiments were conducted with increasing doses of fisetin (1 to 10 µM), for a 15 minutes incubation with RANKL. Blots are representative of 3 independent experiments. (C). Raw264.7 were transfected with NF-κB-luc reporter for 6 hours, pretreated with DMSO as control (fisetin 0 µM) or fisetin (1 to 5 µM) for 3 hours then with RANKL and DMSO as control (fisetin 0 µM) or fisetin (1 to 5 µM) for 48 additional hours before RLU measurement. RLU was related to the total protein concentration for each point. (n = 3 wells, representative of 3 independent experiments). (D). Raw264.7 were pretreated with DMSO as control (fisetin 0 µM) or fisetin (5 µM) for 3 hours, then induced with RANKL and DMSO as control (fisetin 0 µM) or fisetin (5 µM) for 6 hours and the indicated mRNAs were analyzed by RT qPCR. (n = 3 wells, representative of 3 independent experiments). For all data, (*) significantly different from control, p<0.05, (#) significantly different from RANKL-fisetin 0 µM, p<0.05.

NF-κB is a key signaling pathway implicated in the early stages of osteoclast differentiation induced by RANKL [2], [29]. In Raw264.7, as expected, RANKL induced all the steps of NF-κB signaling activation: IκB Kinase α/β ( (IKKα/β) phoshorylation on serines 176/180, Inhibitor of κbα (IκBα)) phosphorylation on serines 32/36 leading to its degradation and p65 phosphorylation on serine 536 (Fig. 4A). All these events were repressed by the presence of fisetin (Fig. 4A), in a dose dependent manner (Fig. 4B). We thus investigated the effect of fisetin on an NF-κB-dependent reporter gene in Raw264.7 induced by RANKL. The relative light units (RLU) induction by RANKL was clearly lowered by fisetin with a significant effect from 2.5 µM (Fig. 4C). To confirm the inhibitory effect of fisetin on the NF-κB system, we analyzed NF-κB target genes induced by RANKL. As a matter of fact, IκBα and the chemokines RANTES, monocyte chimoattractant protein 1 (MCP-1) and macrophage inflammatory protein 1 alpha (MIP-1α)) mRNAs were induced by 2.5 to 4 fold by RANKL (Fig. 4D); the induction was significantly lower in the presence of fisetin. These results imply that fisetin is able to repress osteoclast differentiation by counteracting RANKL-induced NF-κB signaling.

### Fisetin Represses RANKL-induced p38 MAPK/JNK Signaling and c-Fos/NFATc1 Expression

**Figure 5:**
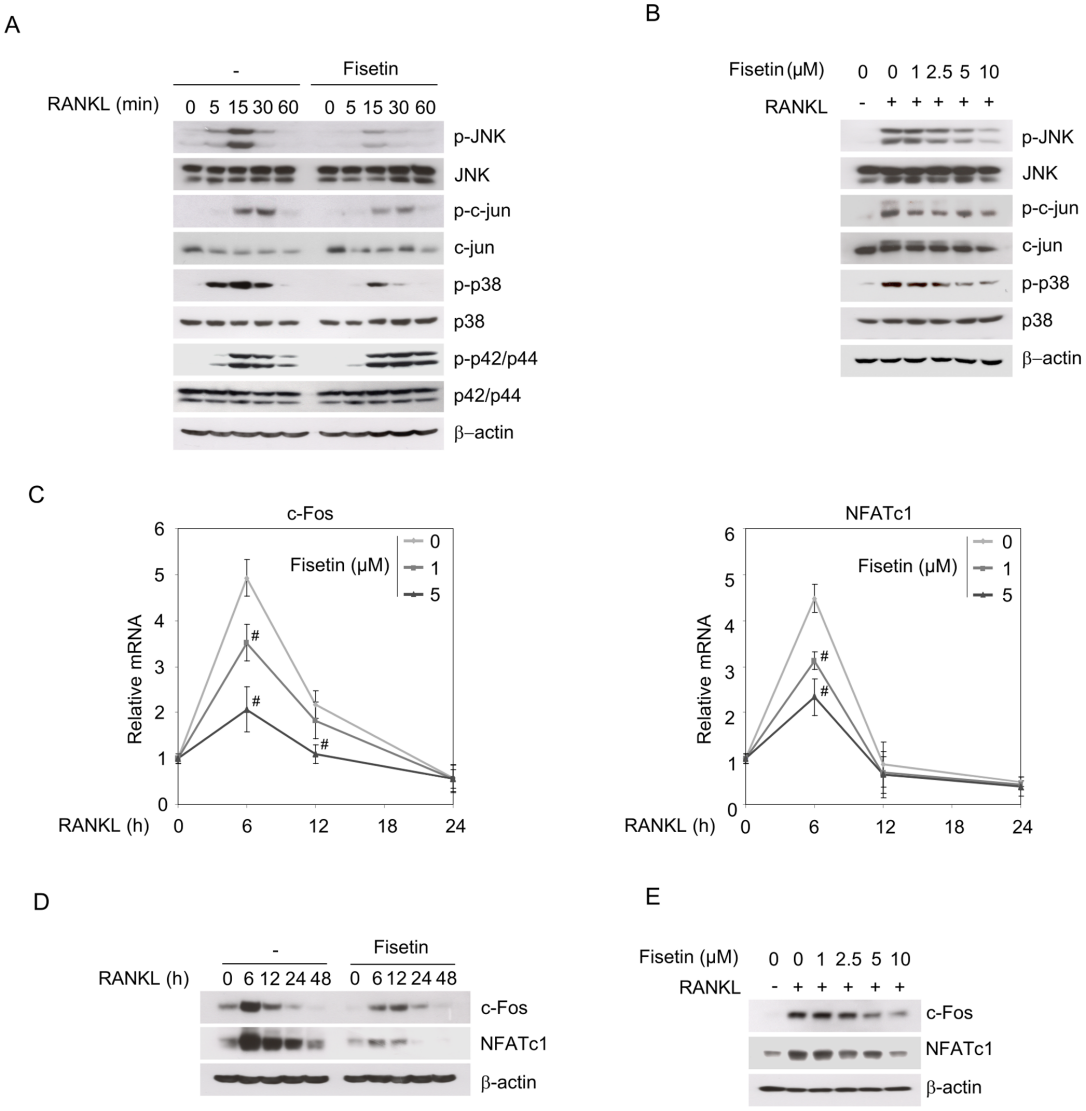
Fisetin represses RANKL-induced p38 MAPK and JNK signaling. (A). Raw264.7 osteoclast precursors were pre-incubated with DMSO as control (−) or fisetin (5 µM) for 3 hours, then induced for 5 to 60 minutes with RANKL together with DMSO as control (−) or fisetin (5 µM). Total protein extracts were analyzed by western-blotting for the indicated proteins. Blots are representative of 3 independent experiments. (B). Similar experiments were conducted with increasing doses of fisetin (1 to 10 µM), for a 15 minutes incubation with RANKL. Blots are representative of 3 independent experiments (C). Raw264.7 were pre-incubated with DMSO as control (fisetin 0 µM) or fisetin (1 to 5 µM) for 3 hours, then induced with RANKL for 6 to 24 hours with DMSO as control (fisetin 0 µM) or fisetin (1 to 5 µM) and the indicated mRNAs were analyzed by RT qPCR. (n = 3 wells, representative of 3 independent experiments). (D). Raw264.7 were pre-incubated with DMSO as control (−) or fisetin (5 µM) for 3 hours, then induced for 6 to 48 hours with RANKL and DMSO as control (−) or fisetin (5 µM) and the indicated proteins were analyzed by western-blotting. Blots are representative of 3 independent experiments. (E). Similar experiments were conducted with increasing doses of fisetin (1 to 10 µM), for a 6 hours incubation with RANKL. Blots are representative of 3 independent experiments. For all data, (*) significantly different from control, p<0.05, (#) significantly different from RANKL-fisetin 0 µM, p<0.05.

We investigated whether fisetin may counteract parallel RANKL-induced pathways involved in osteoclast differentiation such as p38 MAPK, JNK and p42/p44 MAPK [2]. As expected, RANKL treatment induced a transient JNK, c-jun, p38 and p42/p44 phosphorylation revealing their activation (Fig. 5A). The presence of fisetin resulted in a clear lower phosphorylation of JNK, c-jun and p38 (Fig. 5A), in a dose-dependent manner (Fig. 5B). Inversely, we noticed a higher phosphorylated level of p42/p44 when the cells were incubated with fisetin, after 30 and 60 min of RANKL induction (Fig. 5A). In addition to c-jun, c-Fos and NFATc1 are key transcription factors leading to osteoclast differentiation. Actually, c-Fos and NFATc1 mRNAs were both induced after 6 hours of RANKL treatment, while when the cells were cultured in the presence of fisetin, their expression levels were significantly repressed, with a higher effect for the 5 µM dose (Fig. 5C). Fisetin also repressed their protein level after exposure to RANKL (Fig. 5D), in a dose dependent way (Fig. 5E).

### Fisetin Represses Osteoclast Differentiation through MKP-1

**Figure 6:**
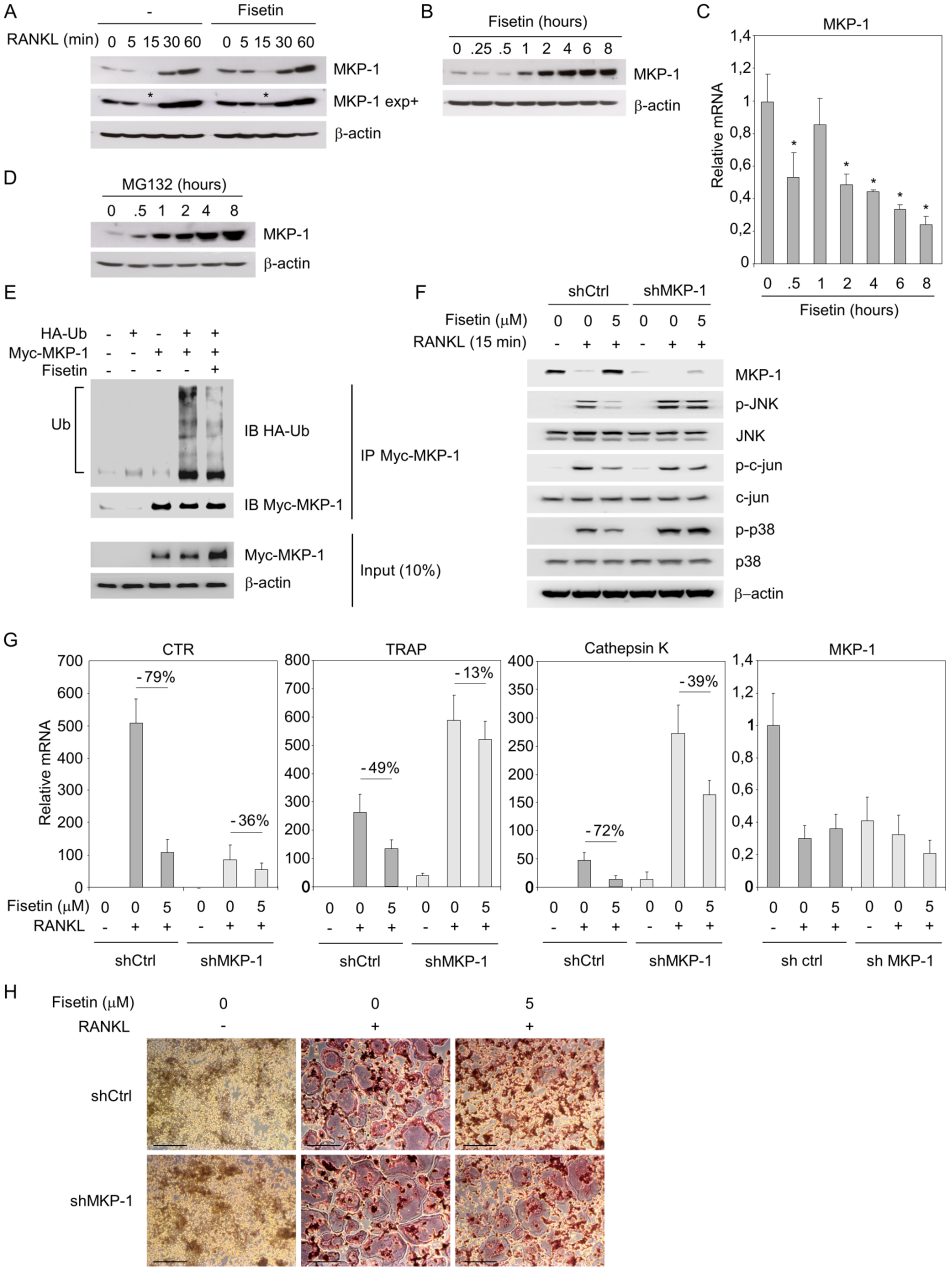
Fisetin represses osteoclast differentiation through MKP-1. (A). Raw264.7 osteoclast precursors were pre-incubated with DMSO as control (−) or fisetin (5 µM) for 3 hours, then induced for 5 to 60 minutes with RANKL and DMSO as control (−) or fisetin (5 µM). Total protein extracts were analyzed by western-blotting for the indicated proteins. MKP-1 exp+ is a long exposure blot that highlights the difference of signal intensities at T15 min (*). Blots are representative of 3 independent experiments. (B). Raw264.7 were incubated with fisetin (5 µM) for 15 minutes to 8 hours, and total protein extracts were analyzed by western-blotting for the indicated proteins. Blots are representative of 3 independent experiments. (C). Raw264.7 were incubated with fisetin (5 µM) for 30 minutes to 8 hours, and MKP-1 mRNA was analyzed by RT qPCR. (*) significantly different from T0, p<0.05. (n = 3 wells, representative of 3 independent experiments). (D). Raw264.7 were incubated with the proteasome inhibitor MG132 (25 µM) for 30 minutes to 8 hours, and total protein extracts were analyzed by western-blotting for the indicated proteins. Blots are representative of 3 independent experiments. (E). Raw264.7 cells were transfected with Myc-MKP-1 and HA-Ub and incubated with DMSO as control (fisetin 0 µM) or fisetin (5 µM) for 4 hours. Whole cell extracts were prepared as described in the section on ubiquitination assays (“Materials and Methods”) and were subjected to immunoprecipitation (IP) with anti-Myc prior western-blotting analysis. Blots are representative of 3 independent experiments. (F). shControl (shCtrl) and shMKP-1 Raw264.7 were pre-incubated with DMSO as control (fisetin 0 µM) or fisetin (5 µM) for 3 hours, then induced for 15 minutes with RANKL and DMSO as control (fisetin 0 µM) or fisetin (5 µM). Total protein extracts were analyzed by western-blotting for the indicated proteins. Blots are representative of 3 independent experiments. (G). shCtrl and shMKP-1 Raw264.7 were pre-incubated with DMSO as control (fisetin 0 µM) or fisetin (5 µM) for 3 hours, then induced for 4 days with RANKL and DMSO as control (fisetin 0 µM) or fisetin (5 µM) and the indicated mRNAs were analyzed by RT qPCR. (n = 3 wells, representative of 3 independent experiments). (H). Similar experiments as in G were conducted and TRAP staining was performed at the end of the differentiation process. Scale bars correspond to 250 µm. (n = 3 wells, representative of 3 independent experiments).

The previous results indicate that fisetin controls the p38 MAPK and JNK signaling pathways, but the principal upstream player mediating fisetin effects remained to be determined. To further elucidate the mechanisms of actions, we studied the expression level of the MAPK Phosphatase-1 (MKP-1), a phosphatase responsible for p38 MAPK and JNK deactivation [30], 31. Interestingly, the Raw264.7 cells that have been pre-incubated with fisetin exhibited a higher level of MKP-1 than the control ones (Fig. 6A, 0 min). Moreover, this effect was higher for all the time points of RANKL induction, notably for the 15 minutes RANKL induction, when MKP-1 level is very low and the p38 MAPK and JNK are highly activated (Fig. 6A, MKP-1 exp+, see (*)). These results suggest that fisetin positively controls the MKP-1 expression level that may lead to a lesser p38 MAPK and JNK activation. A kinetic of fisetin treatment actually revealed its potential to increase MKP-1 protein level (Fig. 6B), although mRNA levels were decreased (Fig. 6C). Thus, we speculated that fisetin may regulate MKP-1 level by controlling its degradation by the proteasome, a molecular complex responsible for proteins breakdown following their conjugation to ubiquitin [32]. In Raw264.7, MKP-1 protein level was found to be dependent on the ubiquitin proteasome system (UPS), as revealed by its stabilization following UPS inhibition by MG132 (Fig. 6D). In this light, we studied whether fisetin might induce a reduction in the extent of conjugation of MKP-1 to polyubiquitin chains which are recognized by the 26S proteasome for degradation. After cotransfection of Myc-MKP-1 with HA-Ub, MKP-1 was immunoprecipitated with anti Myc, and the polyubiquitin chains were revealed with the anti-HA antibody. As shown in Fig. 6E, MKP-1 is efficiently ubiquitinated in control cells. However, when the cells were cultured in the presence of fisetin, the ubiquitination level was clearly decreased. This result shows that fisetin stabilizes MKP-1 by inhibiting its conjugation to ubiquitin chains, thus leading to its lower degradation by the ubiquitin proteasome system. To confirm that the inhibitory action of fisetin on osteoclast differentiation was dependent on MKP-1, the latter was knocked-down in Raw264.7 using lentiviral infections. As expected, the RANKL-activated p38 MAPK and JNKs signaling pathways were inhibited by fisetin in “shControl” cells (shCtrl); p38, JNK and c-jun were less phosphorylated in cells cultured in the presence of fisetin (Fig. 6F). However, fisetin had a lower inhibitory action in “shMKP-1” cells. To study the role of this difference in signaling activities on the osteoclast differentiation, both cells types were induced to differentiate with RANKL for 4 days in absence or presence of fisetin. At the end of the differentiation protocol, fisetin was found to repress the RANKL induced expression of CTR, TRAP and cathepsin K mRNAs in “shCtrl” cells (Fig. 6G). In contrast, in “shMKP-1” cells, fisetin presented a lower repressive activity. Accordingly, only a very few TRAP(+) MNC were present in “shCtrl” cells induced to differentiate by RANKL in the presence of fisetin, while a large number of giant TRAP(+) MNC were formed in “shMKP-1” cells (Fig. 6H). These experiments finally demonstrate that fisetin represses osteoclast differentiation, in part, through MKP-1.

## Discussion

Current drugs used for the treatment of osteoporosis may exert adverse side effects as jaw osteonecrosis or upper gastrointestinal diseases for bisphosphonates [33], [34] and increased risks of endometrial cancer for selective estrogen receptor modulators (SERMs) [35]. Therefore, naturally occurring bioactive dietary compounds endowed with positive effects on bone health represents an attractive alternative for managing osteoporosis. In this study, we show that the consumption of fisetin, a polyphenol found in plants and fruits, prevents bone loss induced by estrogen-privation or inflammation in mice. Although fisetin belongs to flavonoid polyphenols, whose some members are qualified as phytoestrogens, several studies have demonstrated its very low hormonal activity in estrogen sensitive cells, as compared to others flavonoids [36], [37]. In our experimental conditions, the beneficial action on bone tissue was probably not related to a phytoestrogenic activity, as supported *in vivo* by a non-uterotrophic effect in ovariectomized mice (Fig. 1B).

Both *in vivo* and *in vitro*, we have demonstrated that fisetin exerts anti-inflammatory activities. In mice, the induction of inflammatory parameters by LPS injections where counteracted by fisetin: we observed a return to the basal level of serum sTNFR1 level, the spleen and the thymus weight as well. Fisetin has already been described as an anti-inflammatory agent in LPS or ovalbumin-induced pulmonary inflammation [38], [39] and in collagen-induced arthritis [21] in mice. Associated molecular mechanisms relied on an inhibition of the NF-κB system as already described *in vitro* in LPS treated macrophages [22] or TNFα treated cancer cells [40]. Consistently, we demonstrated that fisetin inhibited the RANKL-induced NF-κB signalling and transcriptional activity, as observed for the targeted inflammatory chemokines RANTES, MCP-1 and MIP-1α. Furthermore, it has been shown that NF-κB regulates the transcription of NFATc1 through p50 and p65 binding to its promoter [12], while an inhibitor of NF-κB represses its expression [13]. Thus, the down-regulation of NF-κB system and the subsequent inhibition of RANKL-induced NFATc1 expression contribute to explain the inhibitory effect of fisetin on osteoclastogenesis.

While we were preparing the manuscript, Choi et al. [41] and Sakai et al. [42] published their work about fisetin action on osteoclast differentiation. As demonstrated in our study, they both show that fisetin dose-dependently inhibits the osteoclast differentiation by repressing the RANKL-induced c-Fos and NFATc transcription factors and osteoclasts markers expressions, thus corroborating our results on the potential of fisetin on osteoclastogenesis. Regarding the signalling pathways inhibition, our results on p38 are consistent with Choi et al. data while those on JNK parallel with Sakai et al. and previous studies performed in prostate and fibroblast-like synovial cells [21], [43]. The discrepancy on NF-κB signalling between Sakai et al. and us may be explained by differences in the experimental protocol: our pre-incubation time with fisetin was shorter (3 vs 12 hours) and fisetin was still present with RANKL in our experiments. To date, in our manuscript, the findings on steoclasts and bone physiology modulation by fisetin are strengthened by *in vivo* results.

In order to better characterize the molecular mechanisms by wich fisetin controls MAPK-regulated osteoclastogenesis, we studied its capacity to control the phosphatase MKP-1, an upstream modulator of p38 and JNK activities. MKP-1 exerts critical functions in a large number of physiological and pathophysiological processes. It is a negative regulator of innate and adaptative immunity, it plays an important role in metabolism, potentially a pathophysiological role in the progression of obesity and metabolic syndrome and is a regulator of bone mass as well [31], [44]. Actually, the lack of MKP-1 is associated with a reduced trabecular bone density in female mice [45], [46]. *In vivo*, Carlson et al. demonstrated that MKP-1 negatively regulates osteoclast differentiation and activation in response to LPS injection. In MKP-1^−/−^ primary macrophages, the p38 MAPK and JNK were more activated in response to RANKL than in MKP-1^+/+^ one. Moreover, following M-CSF and RANKL induction, the osteoclast resorbing activity was higher in the knock-out macrophages than in wild-type. Thus, the authors conclude that MKP-1 negatively regulates osteoclast differentiation and activation by dephosphorylating p38 MAPK and JNK, two molecules that play key roles in the differentiation and activation of osteoclasts. These results are clearly in accordance with our findings showing that fisetin represses osteoclast differentiation and activity in part by increasing the MKP-1 protein level, and thus repressing the RANKL-induced activation of p38 MAPK and JNK. One remaining divergent point concerns the fact that in response to RANKL, MKP-1^−/−^ spleen-derived macrophages formed fewer and smaller osteoclasts compared to MKP-1^+/+^ one; we actually observed a reduced number of osteoclasts using BMC and Raw264.7 when the level of MKP-1 was high (in the presence of fisetin) and that shMKP-1 knockdown did not significantly modified the osteoclasts phenotype. We can speculate that this difference is associated with the diverging endogenous level of MKP-1 (knock-down versus knock-out). Finally, in our study, we show that fisetin acts by stabilizing MKP-1 through a decrease of its ubiquitination level. Such a regulation of MKP-1 by the ubiquitin-proteasome pathway has already been described in fibroblasts in which the activation of p42 and p44 MAPK (ERK1/2) leads to MKP-1 phosphorylation and subsequent stabilization [47]. Then, it is of major interest to note that RANKL activativation of p42/p44 was increased in preosteoclasts treated with fisetin (Fig. 5A). Such a regulation of ERK activity by fisetin is consistent with previous studies from the field of neurodegenerative diseases [19], [20]. In this context, ERK activation by fisetin may induce MKP-1 phosphorylation and stabilization which in turn, will be responsible of a lower activation of p38 and JNK in response to RANKL.

**Figure 7:**
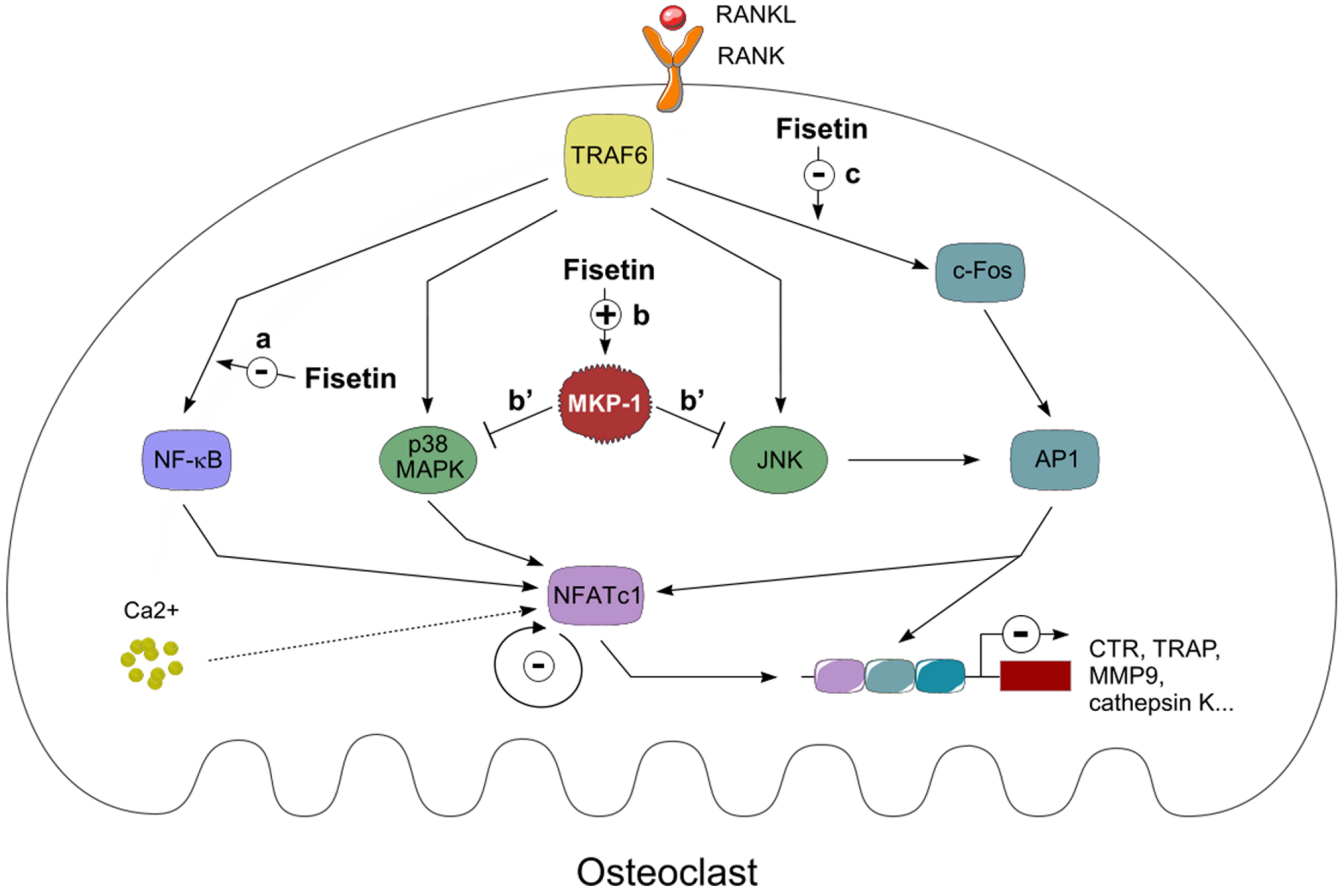
Molecular mechanisms by which fisetin controls osteoclast differentiation and activity. Fisetin negatively controls osteoclasts differentiation process by inhibiting RANKL-induced NF-κB signaling (a), stabilizing MKP-1 (b), the phosphatase that negatively controls p38 MAPK and JNK signaling pathways, thus inhibiting their RANKL-induced activation (b’) and counteracting the RANKL-induced c-Fos expression (c). These actions result in a transcriptional repression of the key transcription factor NFATc1 and a subsequent inhibition of its target genes: CTR, TRAP, MMP9 or cathepsin K.

To conclude, our results demonstrate that a natural product, namely fisetin, positively modulates bone physiology and prevents estrogen deficiency and inflammation-induced osteoporosis. We have shown that it exerts its effect by repressing osteoclast differentiation process (Fig. 7), both *in vivo* and *in vitro*. Therefore, fisetin should be further considered as a potential nutritional target to improve bone health and a promising alternative in the design of new opportunities for the treatment of bone diseases including osteoporosis.
